# 9+2 to 9+0 axoneme conversion in *Leishmania*

**DOI:** 10.1186/2046-2530-4-S1-O5

**Published:** 2015-07-13

**Authors:** R Wheeler, K Gull, E Gluenz

**Affiliations:** 1 Max Planck Institute for Cell Biology and Genetics, Dresden, Germany; 2 Sir William Dunn School Of Pathology, University of Oxford, Oxford, UK

## 

Eukaryotic cilia/flagella typically exhibit two characteristic ultrastructures reflecting two main classes of function; a 9+2 axoneme for motility, and a 9+0 axoneme for sensation/signalling. The key difference between these two ultrastructures is whether a central pair is nucleated at the basal plate of the centriole/basal body transition zone. We aimed to determine whether basal bodies are precommitted to nucleate one ultrastructure of cilium or can nucleate either, and whether transitions between the two ultrastructures of cilia post-assembly is possible.

We analysed axoneme ultrastructure change using *Leishmania mexicana*, a unicellular eukaryotic parasite which has a long motile 9+2 flagellum in the sand fly vector and a short immotile 9+0 flagellum when infecting mammalian macrophages. Using parasites expressing GFP proteins marking different axonemal structures, light and electron microscopy we analysed the transformation of axoneme ultrastructure by direct observation during macrophage infection and *via *two in vitro protocols.

This analysis showed that immature pro-basal bodies are 'bipotent' and are not committed to form either a 9+2 or 9+0 axoneme; that during the maturation process they become determined to one pathway of assembly. Axoneme ultrastructure was also flexible, 9+0 axonemes can form by both *de novo *extension from BBs and restructuring of existing 9+2 axonemes by removal of the central pair.

